# The hodograph equation for slow and fast anisotropic interface propagation

**DOI:** 10.1098/rsta.2020.0324

**Published:** 2021-09-06

**Authors:** P. K. Galenko, A. Salhoumi

**Affiliations:** ^1^ Friedrich-Schiller-Universität-Jena, Faculty of Physics and Astronomy, Otto Schott Institute of Materials Research, 07743 Jena, Germany; ^2^ Department of Theoretical and Mathematical Physics, Laboratory of Multi-Scale Mathematical Modeling, Ural Federal University, Ekaterinburg 620000, Russian Federation; ^3^ University of Hassan II Casablanca, Faculty of Sciences Ben M’Sik, Department of Physics, Laboratory of Condensed Matter Physics (LPMC), BP 7955 Casablanca, Morocco

**Keywords:** interface, anisotropy, phase field, growth, model

## Abstract

Using the model of fast phase transitions and previously reported equation of the Gibbs–Thomson-type, we develop an equation for the anisotropic interface motion of the Herring–Gibbs–Thomson-type. The derived equation takes the form of a hodograph equation and in its particular case describes motion by mean interface curvature, the relationship ‘velocity—Gibbs free energy’, Klein–Gordon and Born–Infeld equations related to the anisotropic propagation of various interfaces. Comparison of the present model predictions with the molecular-dynamics simulation data on nickel crystal growth (obtained by Jeffrey J. Hoyt *et al.* and published in *Acta Mater.* **47** (1999) 3181) confirms the validity of the derived hodograph equation as applicable to the slow and fast modes of interface propagation.

This article is part of the theme issue ‘Transport phenomena in complex systems (part 1)’.

## 1. Introduction

**Figure 1.**
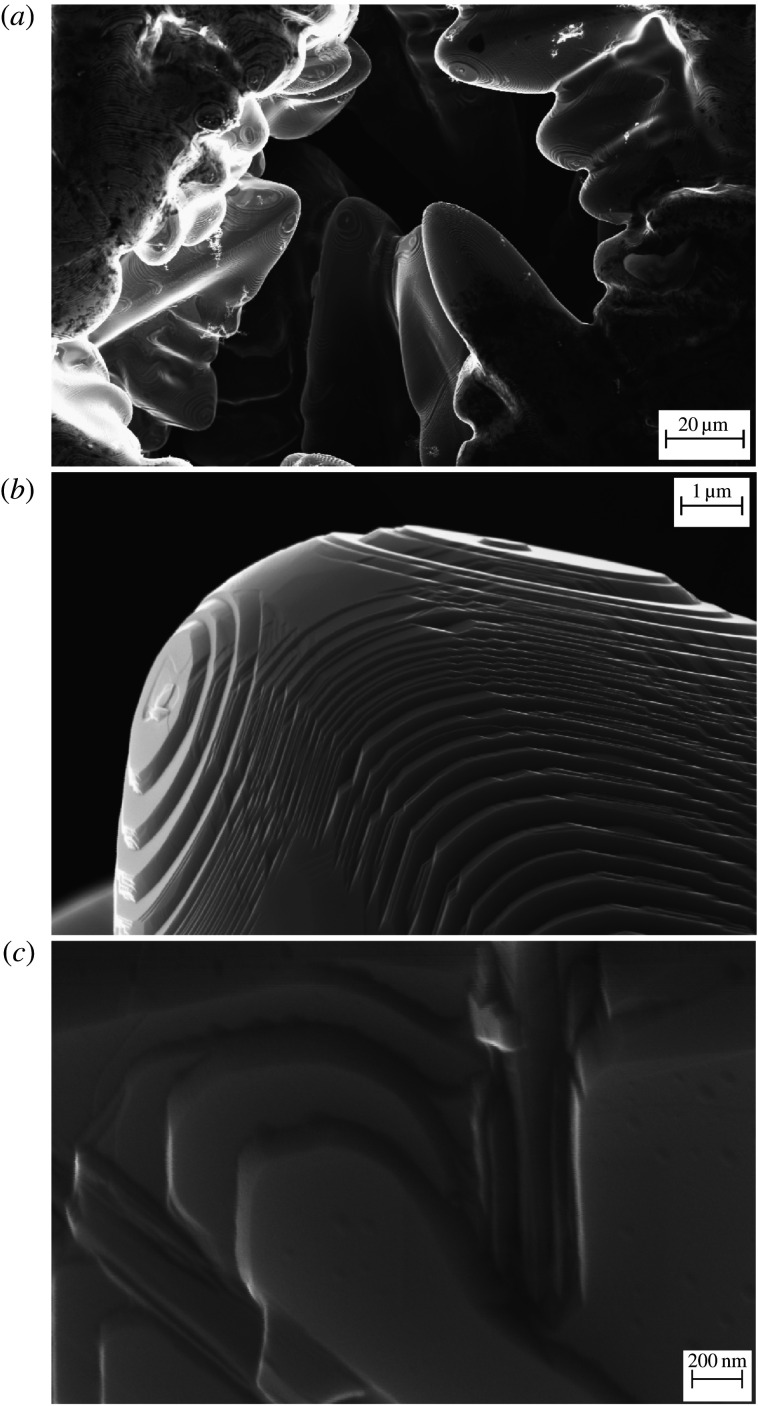
Anisotropic interfaces of crystals in solidified Ni-Cu droplet processed in electromagnetic levitation facility [5]. (*a*) Dendritic patterns. (*b*) Region around the tip of one of the dendrites with clearly visible steps. (*c*) Visible steps at the dendrite surface at higher resolution.

Anisotropy of interfaces plays a crucial role in the formation of equilibrium shapes [1], changing of growth direction of crystals to the preferable one at a critical governing parameter [2], selection of stable mode of dendritic growth [3] and facetting with the formation of microscopic defects in the bulk of crystals [4]. Figure 1 shows a region around a dendritic tip, the growth of which has been selected and faceted due to the existence of anisotropy of solid–liquid interface. From the near equilibrium properties, these anisotropic interfaces lead to crystallographically oriented properties such as free energy, magnetization and surface tension [5]. From the kinetic properties, the interface mobility and coefficients of atomic attachment to the interface strongly depend on the crystallographic faces orientation [6,7].

In the last century there has been great success in the description of equilibrium shapes and their motion. Herring [8] generalized the formalism of Wulff on thermodynamics of crystal surfaces. Cahn & Hoffman [9,10] introduced a useful construction, the so-called ξ-vector, to characterize equilibrium shapes and missing orientations of sharp interfaces. Caginalp and then Kobayashi [11–13] were the first to introduce the anisotropy in the diffuse interface formalism with the surface tension anisotropy by allowing the gradient energy factor to be dependent on the orientation of the phase interface. Wheeler & McFadden [14–16] provided description of anisotropic interfaces using the Cahn–Hoffman ξ-vector within the framework of the phase field model. Avoiding unstable interfacial orientations, the growth of highly anisotropic interfaces with facetting and edges was analysed by Eggleston *et al.* [17] and Debierre *et al.* [18]. Finally, an overview by Sekerka [19] presented approaches in the description of anisotropic crystal growth.

In the present work, we extend the description of anisotropic interfaces to the highly rapid regimes of their motion with the appearance of local non-equilibrium effects [20]. With this aim, a kinetic phase field model [21,22], which would be reduced to the single equation of motion called ‘the hodograph equation of interface’, is used [23,24]. The hodograph equation predicts non-stationary regimes as well as steady-state regimes of interface motion and it has been applied, for instance, to quantitative estimations of non-stationarity periods of dendrite growth [25]. Using the formalism of the Cahn–Hoffman ξ-vector and the presently developed anisotropic phase field model, we follow the analysis of Wheeler & McFadden [14] who obtained the sharp interface limit corresponding to the case for which the diffuse interface width is small compared to a characteristic macroscopic length scale.

The article is organized as follows. As an advancement of the isotropic phase field model (§2) we introduce the kinetic phase field model with crystalline anisotropy in its hyperbolic formulation (§3). The obtained hodograph equation in the form of Herring–Gibbs–Thomson-type equation is compared with the previous equations of equilibrium, acceleration-velocity-dependent equation as well as Born–Infeld and Klein–Gordon equations describing the propagation of isotropic and anisotropic interfaces (§4). A quantitative comparison of linear and nonlinear equations for interface motion which follow from the obtained kinetic equation for anisotropic crystalline interface is given (§5). A summary of our conclusions is presented (§6). Finally, electronic supplementary material, [26] and appendix A add the material for the derivation of the present hodograph equation applicable to the slow and fast modes of interface propagation.

## 2. Isotropic phase field equation

The isotropic phase field equation and its solution were obtained in the work [23] and analysed in comparison with MD-data in [27]. In this section, we recollect the results of these works necessary to reproduce anisotropic advancement of the hyperbolic phase field model.

The free energy functional for the entire system of the volume $v_{0}$ in the isotropic case can be written in the form

2.1 $$\tilde{G}=\int_{v_{0}}\left\{\frac{1}{2}\varepsilon_{\phi }^{2}|\nabla \phi {|}^{2}+G\left(T,C,\phi ,\frac{\partial \phi }{\partial t}\right)\right\}\text{d}v_{0},$$

which yields the dimensional isotropic form of the dynamic equation as

2.2 $$\tau_{\phi }\frac{\partial^{2}\phi }{\partial t^{2}}+\frac{\partial \phi }{\partial t}=M_{\phi }\varepsilon_{\phi }^{2}\nabla^{2}\phi -M_{\phi }\Delta G\frac{\text{d}p(\phi )}{\text{d}\phi }-M_{\phi }W_{\phi }(T,C)\frac{\text{d}g(\phi )}{\text{d}\phi }.$$

Here, ϕ is the phase field variable defining the phase state as

2.3 $$\phi =\left\{ \begin{array}{ll} 0, & \text{in the liquid phase},\\ 1, & \text{in the solid phase}, \end{array}\right.$$

$\tau_{\phi }$ is the relaxation time of the gradient flow for the phase field ∂ϕ/∂t, $M_{\phi }$ is the mobility, C is the solute concentration and T is the temperature.

The Gibbs free energy change on transformation ΔG takes into account direction of transformation

2.4 $$\Delta G=G_{s}(T,C)-G_{l}(T,C)\left\{ \begin{array}{ll} <0, & \text{solidification},\\ >0, & \text{melting}, \end{array}\right.$$

and the variety of transformations obtained for enthalpy of fusion, $\Delta H_{f}(T)$ [28], dilute mixtures [29], and functions obtained from thermodynamics databases [30]. Note that $G_{i}(T,C)$(i=l,s) is the Gibbs free energy of the phase, and the indexes l and s are related to the liquid and solid phases, respectively.

The interpolation function p(ϕ) and the double-well function g(ϕ) are defined by [31]

2.5 $$p(\phi )=(3-2\phi )\phi^{2}\;\text{and}\;g(\phi )={\left(1-\phi \right)}^{2}\phi^{2}.$$

According to definition (2.3), the interpolation function p(ϕ) varies monotonically from p(0)=0 to p(1)=1.

The gradient energy coefficient $\varepsilon_{\phi }$ in equation (2.2) is related to the interface energy γ at equilibrium, ΔG=0, $G_{s}(T,C)=G_{l}(T,C),$ for which equation (2.2) admits one-dimensional steady solution at ∂ϕ/∂t=0:

2.6 $$\phi (x)=\frac{1}{2}\left[1-\tanh\left(\frac{x}{\delta }\right)\right],$$

with the diffuse interface stationary width

2.7 $$\delta =\frac{\sqrt{2}\varepsilon_{\phi }}{\sqrt{W_{\phi }(T,C)}},$$

and under the boundary conditions ϕ=1 as x≤δ and ϕ=0 as x≥δ. Then, the surface energy γ of the isotropic interface is given by

2.8 $$\begin{array}{rl} \gamma & =\int_{-\infty }^{+\infty }\left[\frac{\varepsilon_{\phi }^{2}}{2}{\left(\frac{\text{d}\phi }{\text{d}x}\right)}^{2}+W_{\phi }(T,C)g(\phi )\right]\text{d}x\\ & =\varepsilon_{\phi }^{2}\int_{-\infty }^{+\infty }{\left(\frac{\text{d}\phi }{\text{d}x}\right)}^{2}\;\text{d}x=\frac{\varepsilon_{\phi }\sqrt{W_{\phi }(T,C)}}{3\sqrt{2}}=\frac{\delta W_{\phi }(T,C)}{6}, \end{array}$$

which is proportional to the product of the interface width δ and the energy per unit volume associated with the barrier height $W_{\phi }$.

In the dynamics, $\Delta G=G_{s}(T,C)-G_{l}(T,C)\neq 0$, equation (2.2) admits one-dimensional travelling-wave solution [23,32]

2.9 $$\phi (x,t)=\frac{1}{2}\left[1-\tanh\left(\frac{x-Vt}{\ell }\right)\right],$$

with the boundary conditions ϕ→1 as x−Vt→−∞ and ϕ→0 as x−Vt→+∞, with the constant velocity V limited by $V_{\phi }$ as a maximum speed of phase field propagation,

2.10 $$V=\pm \frac{\mu (-\Delta G)}{\sqrt{1+{\left(\mu \Delta G/V_{\phi }\right)}^{2}}}<|V_{\phi }|,\;V_{\phi }={\left(\frac{M_{\phi }\varepsilon_{\phi }^{2}}{\tau_{\phi }}\right)}^{1/2}={\left(\frac{D_{\phi }}{\tau_{\phi }}\right)}^{1/2},$$

the velocity-corrected effective interface thickness,

2.11 $$\ell =\frac{2\delta }{3}{\left[1-{\frac{V^{2}}{V_{\phi }}}^{2}\right]}^{1/2},$$

and the mobility $M_{\phi }$ related to the isotropic interface mobility μ as

2.12 $$\mu =\frac{18\gamma }{W_{\phi }(T,C)}M_{\phi }(T).$$

First, the particular solution (2.9)–(2.12) with the hyperbolic tangent function follows from the general set of analytical solutions of Allen–Cahn-type equations [33] which is given by equation (2.2). Second, the interface velocity, V, cannot exceed the maximum speed of disturbance propagation in the phase field, because the phase field itself dictates the interface shape and its velocity, i.e. $V<V_{\phi }$ in the solutions to equations (2.9)–(2.12). Third, with regard to the effective interface thickness (2.11), one has to note two important issues: (i) with increasing interface velocity, ℓ should become smaller than the constant interface width δ that has been chosen as a reference for the interface thickness in equilibrium state, equation (2.7); (ii) within the limit $V\to V_{\phi }$, one gets ℓ→0, therefore, the phase field variation will be steeper with the tendency to build up a sharp interface as the velocity increases.

In the dimensionless isotropic form, equation (2.2) can be re-written taking into account equations (2.8) and (2.12) as

2.13 $$\begin{array}{rl} & \frac{18}{\mu }\left(\frac{ℜ}{\Delta H_{f}}\right)\left(\frac{\Delta H_{f}}{W_{\phi }(T,C)}\right)\left(\frac{\gamma }{\Delta H_{f}ℜ}\right)\left[\tau_{\phi }\frac{\partial^{2}\phi }{\partial t^{2}}+\frac{\partial \phi }{\partial t}\right]\\ & \;=18\left(\frac{\Delta H_{f}}{W_{\phi }(T,C)}\right){\left(\frac{\gamma }{\Delta H_{f}ℜ}\right)}^{2}{ℜ}^{2}\nabla^{2}\phi -\frac{W_{\phi }(T,C)}{\Delta H_{f}}\frac{\text{d}g(\phi )}{\text{d}\phi }-\frac{\Delta G}{\Delta H_{f}}\frac{\text{d}p(\phi )}{\text{d}\phi }. \end{array}$$

To obtain the Herring–Gibbs–Thomson-type equation following from the hyperbolic phase field equation (2.13), we shall obtain the sharp interface limit corresponding to the case that the diffuse interface width is small compared to a characteristic macroscopic length scale ℜ, which can be taken to represent a typical radius of interfacial curvature in the non-planar case [14]. More specifically, we consider the distinguished limit

2.14 $$\frac{\varepsilon_{\phi }}{2\sqrt{W_{\phi }(T,C)}ℜ}\to 0\;\text{and}\;\frac{\Delta H_{f}}{4W_{\phi }(T,C)}\to 0,$$

while maintaining a finite value for the ratio

2.15 $$\frac{\varepsilon_{\phi }}{\sqrt{W_{\phi }(T,C)}}\equiv \text{finite},$$

in order that the surface energy remains finite in the sharp interface limit. Using these scales, it is convenient to choose the length and coordinates in units of ℜ, the energy density ΔG and energy barrier height $W_{\phi }$ in unit of latent heat $\Delta H_{f}$, the time t and relaxation time $\tau_{\phi }$ in units of a diffusive time scale τ consistent with the accompanying energy diffusion processes, the surface energy γ in units of $\Delta H_{f}ℜ$ and the interface kinetics given by μ in units of $ℜ/(\Delta H_{f}\tau )$. Then, the isotropic form of equation (2.2) follows from equation (2.13) as

2.16 $$72\frac{\gamma }{\mu }\epsilon \left[\tau_{\phi }\frac{\partial^{2}\phi }{\partial t^{2}}+\frac{\partial \phi }{\partial t}\right]=72\epsilon \gamma^{2}\nabla^{2}\phi -\frac{1}{4\epsilon }\frac{\text{d}g(\phi )}{\text{d}\phi }-\Delta G\frac{\text{d}p(\phi )}{\text{d}\phi },$$

where ϵ plays the role of a small parameter in the subsequent asymptotic treatment and it is defined by

2.17 $$\epsilon =\frac{\Delta H_{f}}{4W_{\phi }(T,C)}.$$

This ratio means that the high value of barrier $W_{\phi }(T,C)$ between phases provides a smallness of ϵ and the asymptotic limit ϵ→0.

## 3. Hyperbolic phase field model with anisotropy

### (a) Free energy and scaled phase field equation

Consider a binary system consisting of solvent and solute under isothermal condition, with the temperature T being constant in the overall system. The system is undergoing phase transition, solidification/melting, from the undercooled/overheated state. Taking the existence of the anisotropic diffuse solid–liquid interface into account [34,35], the free energy functional in units of $\Delta H_{f}$ for entire system of the volume $v_{0}$ is described as

3.1 $$G=\int_{v_{0}}\left\{\frac{\gamma^{2}(n)}{2}|\nabla \phi {|}^{2}+G\left(T,C,\phi ,\frac{\partial \phi }{\partial t}\right)\right\}\text{d}v_{0},$$

where length, time, Gibbs potential, G(T,C,ϕ,∂ϕ/∂t) and homogeneous functions of surface energy, γ(n), are measured in units of ℜ, τ, $\Delta H_{f}$ and $\Delta H_{f}ℜ$, respectively, and γ is the surface energy of the interface depending on the normal vector n pointing from solid to liquid,

3.2 $$n=-\frac{\nabla \phi }{|\nabla \phi |}.$$

The homogeneous extension of γ(n) is given by [36]

3.3 $$|\nabla \phi |\gamma \left(-\frac{\nabla \phi }{|\nabla \phi |}\right)=\gamma (-\nabla \phi ),$$

such that the free energy functional (3.1) becomes

3.4 $$G=\int_{v_{0}}\left\{\frac{1}{2}{\left[\gamma (-\nabla \phi )\right]}^{2}+G\left(T,C,\phi ,\frac{\partial \phi }{\partial t}\right)\right\}\text{d}v_{0},$$

where the Gibbs potential G(T,C,ϕ,∂ϕ/∂t) is described by the expression [37,38]

3.5 $$G(T,C,\phi )=G_{eq}(T,C,\phi )+G_{\text{neq}}\left(T,\frac{\partial \phi }{\partial t}\right),$$

which has the local equilibrium contribution

3.6 $$G_{eq}(T,C,\phi )=[1-p(\phi )]G_{l}(T,C)+p(\phi )G_{s}(T,C)+W_{\phi }(T,C)g(\phi ),$$

and the local non-equilibrium contribution

3.7 $$G_{\text{neq}}\left(T,C,\frac{\partial \phi }{\partial t}\right)=\frac{\alpha_{\phi }(T,C)}{2}{\left(\frac{\partial \phi }{\partial t}\right)}^{2}.$$

In addition to the interpolation function p(ϕ) and the double-well function g(ϕ) given by equation (2.5), the contributions (3.6) and (3.7) include: the phenomenological coefficients $\alpha_{\phi }(T,C)$ proportional to the relaxation time $\tau_{\phi }$ of the gradient flow ∂ϕ/∂t, the barrier $W_{\phi }(T,C)$ between phases as well as the Gibbs free energies $G_{l}(T,C)$ and $G_{s}(T,C)$.

A stable evolution of the entire system is given by the Lyapunov condition of non-positive change of the total Gibbs free energy. For the functional (3.1), this condition gives the inequality

3.8 $$\begin{array}{rl} \frac{\text{d}G}{\text{d}t} & =\int_{v_{0}}\frac{\partial }{\partial t}\left\{\frac{1}{2}{\left[\gamma (-\nabla \phi )\right]}^{2}+G(T,C,\phi ,\partial \phi /\partial t)\right\}\text{d}v_{0}\\ & =\int_{v_{0}}\gamma (-\nabla \phi )\frac{\partial \gamma (-\nabla \phi )}{\partial t}\text{d}v_{0}+\int_{v_{0}}\frac{\partial G(T,C,\phi ,\partial \phi /\partial t)}{\partial t}\text{d}v_{0}\leq 0. \end{array}$$

from which one finds the following phase field equation (appendix A)

3.9 $$\tau_{\phi }\frac{\partial^{2}\phi }{\partial t^{2}}+\frac{\partial \phi }{\partial t}=M_{\phi }\nabla \cdot [\gamma (-\nabla \phi )\xi ]-M_{\phi }\left[\Delta G\frac{\text{d}p(\phi )}{\text{d}\phi }+W_{\phi }(T,C)\frac{\text{d}g(\phi )}{\text{d}\phi }\right].$$

Note that $M_{\phi }$ and ξ are measured in units of ${\left(\Delta H_{f}\tau \right)}^{-1}$ and $\Delta H_{f}$, respectively. Here, the ξ-vector of Hoffman & Cahn [9] is described by

3.10 $$\xi =\frac{\partial \gamma (-\nabla \phi )}{\partial (\nabla \phi )},\;\xi (-\nabla \phi )=-\xi (\nabla \phi ),$$

where the second equality in equation (3.10) defines the ξ-vector as a homogeneous function.

### (b) The hodograph equation as a form of the Herring–Gibbs–Thomson-type equation

The anisotropic form of the phase field equation can be written if we assume that the surface energy γ and the interface mobility μ are homogeneous functions of the first degree in order to include their anisotropy, i.e. γ(−∇ϕ)=−γ(∇ϕ) and μ(−∇ϕ)=−μ(∇ϕ), as for ξ-vector equation (3.10). Indeed, taking into account these conditions and using the same treatments from §2 for equation (2.2), one can find that the anisotropic equation (3.9) takes the following form:

3.11 $$72\frac{\gamma (\nabla \phi )}{\mu (\nabla \phi )}\epsilon \left[\tau_{\phi }\frac{\partial^{2}\phi }{\partial t^{2}}+\frac{\partial \phi }{\partial t}\right]=72\epsilon \nabla \cdot (\gamma \xi )-\frac{1}{4\epsilon }\frac{\text{d}g(\phi )}{\text{d}\phi }-\Delta G\frac{\text{d}p(\phi )}{\text{d}\phi },$$

where equation (2.17) holds for the definition of small parameter ϵ. If the inertial term is neglected, $\tau_{\phi }\to 0$ in equation (3.11), the difference in Gibbs free energy is taken as [28]

3.12 $$\Delta G=\frac{\Delta H_{f}(T_{m}-T)}{T_{m}},$$

(with $T_{m}$ the melting temperature) and the equality (3.11) is accounted for, then the anisotropic phase field equation of Wheeler & McFadden [14] is recovered.

Using asymptotic analysis and some necessary derivations (see electronic supplementary material, [26], especially, eqn (83)), one gets

3.13 $$\begin{array}{rl} \left(-\Delta G\right) & =-\frac{1}{\mu (n)}\left[\frac{\tau_{\phi }A_{n}(t)}{{\left[1-W_{n}^{2}(n,t)\right]}^{3/2}}+\frac{V_{n}(t)}{\sqrt{1-W_{n}^{2}(n,t)}}\right]-\frac{\nabla_{S}\cdot \xi (n)}{\sqrt{1-W_{n}^{2}(n,t)}}\\ & \;+\frac{\frac{1}{2}W_{n}^{2}(n,t)}{{\left[1-W_{n}^{2}(n,t)\right]}^{3/2}}\xi (n)\cdot \left(\frac{\nabla_{S}\gamma (n)}{\gamma (n)}+\frac{\nabla_{S}\mu (n)}{\mu (n)}\right), \end{array}$$

where μ(n) is the orientation dependent interface mobility, $V_{n}(t)$ and $A_{n}(t)=\partial V_{n}(t)/\partial t$ stand, respectively, for the velocity and acceleration normal to the interface,

3.14 $$W_{n}(n,t)=\frac{V_{n}(t)}{V_{\phi }(n)},\;V_{\phi }(n)={\left(\frac{\gamma (n)\mu (n)}{\tau_{\phi }}\right)}^{1/2},$$

and the divergency of the ξ-vector is

3.15 $$\nabla_{S}\cdot \xi =-\sum_{i=1,2}\kappa_{i}\left(\gamma (n)+\frac{\partial^{2}\gamma (n)}{\partial \theta_{i}^{2}}\right),$$

with $\theta_{i}$ (i=1,2) being new coordinates depending on curvilinear coordinates from which both principal curvatures $\kappa_{i}$ (i=1,2) can be obtained (see eqns (90)–(93) in electronic supplementary material, [26]). Note that the velocities $V_{n}(t)$ and $V_{\phi }(n)$ are measured in ℜ/τ and the acceleration $A_{n}$ is measured in $ℜ/\tau^{2}$.

In the sharp interface description (see eqns (96)–(97) in electronic supplementary material, [26]), equation (3.13) becomes

3.16 $$\begin{array}{rl} & \tau_{\phi }\frac{A_{n}(t)+D_{n}(t)}{{\left[1-W_{n}^{2}(n,t)\right]}^{3/2}}+\frac{V_{n}(t)}{\sqrt{1-W_{n}^{2}(n,t)}}\\ & \;=\mu (n)\Delta G+\frac{\mu (n)}{\sqrt{1-W_{n}^{2}(n,t)}}\sum_{i=1,2}\kappa_{i}\left(\gamma (n)+\frac{\partial^{2}\gamma (n)}{\partial \theta_{i}^{2}}\right), \end{array}$$

where

3.17 $$D_{n}(t)=\frac{V_{n}^{2}(t)}{2}\sum_{i=1,2}\kappa_{i}[{\left(\frac{1}{\gamma (n)}\frac{\partial \gamma (n)}{\partial \theta_{i}}\right)}^{2}+\frac{1}{\mu (n)\gamma (n)}\frac{\partial \mu (n)}{\partial \theta_{i}}\frac{\partial \gamma (n)}{\partial \theta i}]$$

is the term dependent on the curvatures $\kappa_{i}$, surface energy γ(n), orientation-dependent interface mobility μ(n) and their first order derivatives with respect to $\theta_{i}$. In the particular case of a convex shape having negative principal curvatures $\kappa_{i}$ ($\kappa_{i}<0$ for i=1,2), the term $D_{n}(t)$ plays a role of deceleration.

Equation (3.16) represents the compact form of acceleration-velocity-dependent Herring equation of a moving curved interface for the arbitrary driving force ΔG. In a general case, this equation can be considered as the hodograph equation for the equilibrium state, slow and fast propagation of anisotropic interface.

## 4. Recovering previously derived model equations

### (a) Equilibrium by Herring equation

The classic form of the Herring equation follows directly from equation (3.16) by taking $V_{n}=0$, $A_{n}=0$ and $D_{n}=0$ [8]:

4.1 $$\Delta G=-\kappa_{1}\left(\gamma (n)+\frac{\partial^{2}\gamma (n)}{\partial \theta_{1}^{2}}\right)-\kappa_{2}\left(\gamma (n)+\frac{\partial^{2}\gamma (n)}{\partial \theta_{2}^{2}}\right).$$

This equation also follows from the zero variational derivative from free energy (3.1) with ∂ϕ/∂t=0 [4]. Such a condition defines the minimal thermodynamic potential with respect to the transfer of particles from one phase to the other. Therefore, equation (4.1) describes the shape of anisotropic particle in equilibrium, presenting a balance between a volumetric tendency to exchange and a surface tendency to save a shape of coexisting phases.

### (b) Acceleration-velocity-dependent Gibbs–Thomson equation

In the case of the isotropic phase field model, the surface energy, the interface mobility and the maximum speed of disturbances are independent of the orientation, i.e. γ(n)=γ, μ(n)=μ and $V_{\phi }(n)=V_{\phi }$ have constant averaged values. Equation (3.16) is then simplifying for i=1,2 due to the zero derivatives

4.2 $$\frac{\partial \gamma (n)}{\partial \theta_{i}}=0,\;\frac{\partial^{2}\gamma (n)}{\partial \theta_{i}^{2}}=0,\;\frac{\partial \mu (n)}{\partial \theta_{i}}=0,\;\frac{\partial^{2}\mu (n)}{\partial \theta_{i}^{2}}=0$$

and

4.3 $$V_{\phi }(n)=V_{\phi }=\sqrt{\frac{\mu \gamma }{\tau_{\phi }}}.$$

Taking into account equations (2.7), (2.8) and (2.12) established in the case of the *isotropic model*, the following relations among the model parameters are obtained

4.4 $$\varepsilon_{\phi }^{2}=2\sigma \delta ,\;M_{\phi }=\frac{D_{\phi }}{2\sigma \delta },\;W_{\phi }=\frac{9\sigma }{\delta },\;V_{\phi }=\sqrt{\frac{D_{\phi }}{\tau_{\phi }}}\equiv \sqrt{\frac{\mu \gamma }{\tau_{\phi }}},$$

where we also used equation (4.3). Using solution (2.6), and taking into account equations (2.7), (2.8), (2.12) and (4.4), the time-dependent thickness of the interface ℓ(t) is now given by [23]

4.5 $$\ell (t)=\frac{2}{3}\delta \sqrt{1-W_{n}^{2}(t)},\;W_{n}<1.$$

The inequality in equation (4.5) has the same meaning as for solutions (2.9)–(2.12): because the phase field itself dictates the interface shape and its velocity, the interface velocity cannot exceed the maximum speed of disturbance propagation in the phase field, $W_{n}<1$.

By the above considerations in the isotropic scheme, we recover from equation (3.16) our generalized acceleration-velocity isotropic Gibbs–Thomson equation found in [23] and analysed for (100)-Ni-crystal orientation in [27] with the same parameters,

4.6 $$\frac{\tau_{\phi }A_{n}(t)}{{\left[1-W_{n}^{2}(t)\right]}^{3/2}}+\frac{V_{n}(t)}{\sqrt{1-W_{n}^{2}(t)}}=\frac{D_{\phi }}{\sigma }\Delta G+\frac{D_{\phi }K}{\sqrt{1-W_{n}^{2}(t)}},$$

with the negative curvature $K=\kappa_{1}+\kappa_{2}$, the phase field assumes the value ϕ=1 and ϕ=0 in the solid and liquid phases, respectively. At small and moderate velocity, $W_{n}\ll 1$ and with the absence of the driving force, ΔG=0, equation (4.6) arrives at

4.7 $$A_{n}(t)+\frac{1}{\tau_{\phi }}V_{n}(t)={\left(V_{\phi }\right)}^{2}K.$$

In two dimensions, this equation was used by Gurtin & Podio-Guidugli [39] to explain the evolution of interface possessing effective inertia consistently with experimental data on the oscillation of quantum crystals [40].

### (c) Velocity-dependent Herring equation

In the case of absence of inertial effects, $\tau_{\phi }\to 0$ ($V_{\phi }\to \infty$, $A_{n}=0$) and constant normal velocity $V_{n}=\text{const}$, equation (3.13) reduces to the Herring–Gibbs–Thomson equation in terms of the Cahn–Hoffman ξ-vector that is found by Wheeler & McFadden [14]

4.8 $$\Delta G=\nabla_{S}\cdot \xi +\frac{V_{n}}{\mu (n)},$$

Using equation (3.15), this equation leads to the velocity-dependent Herring equation [41]

4.9 $$\frac{V_{n}}{\mu (n)}-\Delta G=\kappa_{1}\left(\gamma (n)+\frac{\partial^{2}\gamma (n)}{\partial \theta_{1}^{2}}\right)+\kappa_{2}\left(\gamma (n)+\frac{\partial^{2}\gamma (n)}{\partial \theta_{2}^{2}}\right).$$

which describes the interface motion of anisotropic particle due to imposed Gibbs free energy change on transformation, ΔG, and both principal curvatures, $\kappa_{1}$ and $\kappa_{2}$.

A couple of important particular cases can be outlined, which follow from equation (4.9). First, if the driving force is absent ΔG=0 (i.e. with the absence of supersaturation or supercooling), equation (4.9) predicts the interface motion by both principal curvatures

4.10 $$\frac{V_{n}}{\mu (n)}=\kappa_{1}\left(\gamma (n)+\frac{\partial^{2}\gamma (n)}{\partial \theta_{1}^{2}}\right)+\kappa_{2}\left(\gamma (n)+\frac{\partial^{2}\gamma (n)}{\partial \theta_{2}^{2}}\right).$$

The latter effect can be seen as mean curvature flow described by the Allen–Cahn equation [36,42,43] if both principal curvatures are equal to the mean curvature of the surface, i.e. $\kappa =\kappa_{i}$ (i=1,2) and $\theta =\theta_{i}$ (i=1,2). Therefore, equation (4.10) reduces to

4.11 $$\frac{V_{n}}{\mu (n)}=\kappa \left(\gamma (n)+\frac{\partial^{2}\gamma (n)}{\partial \theta^{2}}\right).$$

Second, in the case of planar interface, $\kappa_{1}=\kappa_{2}=0$, a simplest equation of motion,

4.12 $$V_{n}=\mu (n)\Delta G,$$

can be found from equation (4.9). This equation follows from the classic theory of irreversible processes and from traditional phase field model [11,34].

### (d) Born–Infeld equation

One of the specific cases of nonlinear wave propagation can be found from equation (3.16) under the absence of driving force, ΔG=0, and with the infinite orientation-dependent interface mobility, μ(n)→∞

4.13 $$\frac{\gamma (n)}{V_{\phi }(n)}[A_{n}(t)+D_{n}(t)]=[1-W_{n}^{2}(n,t)]\sum_{i=1,2}\kappa_{i}\left(\gamma (n)+\frac{\partial^{2}\gamma (n)}{\partial \theta_{i}^{2}}\right).$$

Equation (4.13) is the anisotropic Born–Infeld equation, which in its isotropic form, $D_{n}(t)=0$ in equation (3.17), is described by

4.14 $$A_{n}(t)=[V_{\phi }^{2}-V_{n}^{2}]K,$$

with $K=\kappa_{1}+\kappa_{2}$ standing for the negative curvature. Equation (4.14) can be directly obtained from equation (4.6) and it is used in nonlinear electrodynamics [44,45]. Such an analogy of nonlinear waves and the fast phase interface propagation can be clarified in forthcoming works.

### (e) Klein–Gordon equation

Accepting $\tau_{\phi }\to \infty$ and $M_{\phi }/\tau_{\phi }\equiv \text{const}$ in equation (3.9), one gets the undamped Klein–Gordon equation

4.15 $$\frac{\partial^{2}\phi }{\partial t^{2}}=\frac{M_{\phi }}{\tau_{\phi }}\left\{\nabla \cdot [\gamma (-\nabla \phi )\xi \;]-W_{\phi }(T,C)\frac{\text{d}g(\phi )}{\text{d}\phi }\right\},$$

extended to the propagation of anisotropic interface. As is known, to describe peculiarities of the first moments of matter formation, the Klein–Gordon equation is used for the inflation stage of the Universe [46]. In this sense, equation (4.15) can be considered as the Klein–Gordon equation for the anisotropic field propagation of the inflation stage of the matter. Using the definition of the ξ-vector of Hoffman & Cahn (3.10), equation (4.15) can be transformed to the isotropic version of the undamped Klein–Gordon equation [23].

A simplest form of the damped Klein–Gordon equation can also be found from equation (3.9) with $\tau_{\phi }\equiv \text{const}$ but within the limit γ(−∇ϕ)→0:

4.16 $$\frac{d^{2}\phi }{dt^{2}}+\frac{1}{\tau_{\phi }}\frac{\text{d}\phi }{\text{d}t}+\frac{M_{\phi }W_{\phi }(T,C)}{\tau_{\phi }}\frac{\text{d}g(\phi )}{\text{d}\phi }=0.$$

This equation describes the oscillatory motion of matter within the action of the potential function g(ϕ). Such a type of front propagation is used for qualitative analysis of the inflation stages of various fields [46].

## 5. Comparison with data of atomistic simulations

In this section, we shall transform from the dimensionless variables and functions (as described in §2, 3 and 4) to the dimension equations and expressions as is given by table 1.

**Table 1.** Notations and dimensions.

parameter	dimension
Latin:	
$D_{\phi }$, phase field diffusion coefficient	${\text{m}}^{2}\;{\text{s}}^{-1}$
G, Gibbs free energy density	${\text{J m}}^{-3}$
$\Delta H_{f}$, enthalpy of fusion	${\text{J m}}^{-3}$
ℓ, velocity-dependent interface thickness	m
$M_{\phi }$, mobility	${\text{m}}^{3}\;{\text{J}}^{-1}\;{\text{s}}^{-1}$
ℜ, spatial scale	m
$\Delta S_{f}$, entropy of fusion	${\text{J mole}}^{-1}\;{\text{K}}^{-1}$
$T_{m}$, melting temperature	K
t, time	s
$V_{n}$, normal interface velocity	${\text{m s}}^{-1}$
$A_{n}$, normal interface acceleration	${\text{m s}}^{-2}$
$V_{\phi }$, maximum speed of the phase field	${\text{m s}}^{-1}$
$v_{0}$, volume of the entire system	${\text{m}}^{3}$
$W_{\phi }$, energetic barrier between phases	${\text{J m}}^{-3}$
Greek:	
γ, interfacial free energy	${\text{J m}}^{-2}$
δ, equilibrium interface thickness	m
$\varepsilon_{\phi }$, gradient energy factor	${\left({\text{J m}}^{-1}\right)}^{1/2}$
κ, interfacial mean curvature	${\text{m}}^{-1}$
$\kappa_{i}$ (i=1,2), principal curvature	${\text{m}}^{-1}$
μ(n), orientation-dependent interface mobility	${\text{m}}^{4}\;{\text{J}}^{-1}\;{\text{s}}^{-1}$
$\mu_{klm}$, interface kinetic coefficient	${\text{m K}}^{-1}\;{\text{s}}^{-1}$
ϕ, phase field variable	—
τ, time scale	s
$\tau_{\phi }$, relaxation time of the gradient flow	s

At small driving force of solidification or melting, interface velocity has a linear dependence on undercooling or overheating, respectively [6] that is also predicted by equation (4.6) in comparison with MD-data [23,24,27]. At large driving force, MD-simulation predicts interface velocity with non-linearity of two types: velocity-undercooling relationship with saturation [47] and with maximum by the crystal growth velocity [48,49]. These nonlinear dependencies in growth kinetics of crystals are well described by the solution of equation (4.6) and by the hyperbolic phase field equation (2.2) [22]. The kinetic undercooling obtained from the steady-state mode predicted by equation (4.6) included in a general undecooling balance at the tip of dendrite allows us to describe the solidification kinetics in glass-forming alloys [50].

### (a) Velocity-driving force relationship

For the planar interface ($\kappa_{1}=\kappa_{2}=0$) propagating with constant velocity ($V_{n}(t)=V_{n}=\text{const}$, A=0, D=0) the hodograph equation (3.16) together with equation (3.14) yields

5.1 $$V_{n}=\pm \frac{\mu (n)(-\Delta G)}{\sqrt{1+{\left[\mu (n)\Delta G/V_{\phi }(n)\right]}^{2}}},\;\mu (n)=\frac{D_{\phi }(n)}{\gamma (n)},$$

where the phase field diffusion coefficient, $D_{\phi }(n)$, and the maximum phase field speed, $V_{\phi }(n)$, are considered as two parameters which define the relaxation time of the gradient flow of the phase field by

5.2 $$\tau_{\phi }=\frac{D_{\phi }(n)}{V_{\phi }^{2}(n)}.$$

With the local equilibrium limit, i.e. with $\tau_{\phi }\to 0$ ($V_{\phi }(n)\to \infty$), equation (5.1) reduces to the linear relation $V_{n}=\mu (n)\Delta G$, which is obtained from the parabolic phase field equation and which can exhibit nonlinear behaviour only due to nonlinearity of the function $D_{\phi }(n)$ (see discussion in [22]).

### (b) Anisotropic functions

By employing cubic harmonics [51,52] and adopting established conventions [53–55], the interfacial free energy γ(n) can be written as

5.3 $$\gamma (n)=\gamma_{0}f(n)=\gamma_{0}f(\epsilon_{1},\epsilon_{2},\epsilon_{3},\ldots ,Q,S),$$

with

5.4 $$f(n)=1+\epsilon_{1}\left(Q-\frac{3}{5}\right)+\epsilon_{2}\left(3Q+66S-\frac{17}{7}\right)+\epsilon_{3}\left(5Q^{2}-16S-\frac{94}{13}Q+\frac{33}{13}\right)+\cdots$$

where $\gamma_{0}$ is the average value of the interfacial free energy, the coefficients $\epsilon_{i}(i=1,2,3)$ are the strengths of anisotropy, and the basic variables Q and S in the context of the normalization take the following form of Fehlner & Vosko [51]:

5.5 $$Q=n_{x}^{4}+n_{y}^{4}+n_{z}^{4}\;\text{and}\;S=n_{x}^{2}n_{y}^{2}n_{z}^{2},$$

where $n_{i}(i=x,y,z)$ are the Cartesian components of the unit normal vector n.

Hoyt *et al.* [56] defined the values of stiffness for different crystallographic orientations in the solid–liquid interface energy needed for parametrization of γ(n). Following the results of their work, the anisotropic function f(n) and its corresponding stiffness $f(n)+d^{2}f(n)/\text{d}\theta^{2}$ can be evaluated around the vanishing angle between the normal n to the interface and the normal to the crystal face, i.e. θ=0 for different orientations. Tables 2 and 3 summarize the obtained results for different orientations, where the first column gives the Miller indices for orientation and parallel direction. Note that the linear system of four interfacial stiffness equations (the middle column in table 3) can be solved using the computed values of interfacial stiffness in MD-simulations from ref. [57].

**Table 2.** Interfacial free energy for different orientations obtained analytically from equation (5.3).

orientation	$\gamma (\text{n})/\gamma_{0}$
100[010];100[001]	$1+\frac{2}{5}\epsilon_{1}+\frac{4}{7}\epsilon_{2}+\frac{4}{13}\epsilon_{3}$
$110[1\bar{1}0];110[001]$	$1-\frac{1}{10}\epsilon_{1}-\frac{13}{14}\epsilon_{2}+\frac{9}{52}\epsilon_{3}$
$111[\bar{1}10];111[11\bar{2}]$	$1-\frac{4}{15}\epsilon_{1}+\frac{64}{63}\epsilon_{2}+\frac{32}{351}\epsilon_{3}$

**Table 3.** Interfacial stiffness for different crystallographic orientations obtained analytically from equation (5.3) (middle column) and the value of linear fitted interfacial stiffness in MD simulations [57] (right column) for Ni.

orientation	$(\gamma +(d^{2}\gamma /d\theta^{2}))/\gamma_{0}$	$\gamma +(d^{2}\gamma /d\theta^{2})$
100[010];100[001]	$1-\frac{18}{5}\epsilon_{1}-\frac{80}{7}\epsilon_{2}-\frac{140}{13}\epsilon_{3}$	0.177
$110[1\bar{1}0]$	$1+\frac{39}{10}\epsilon_{1}+\frac{155}{14}\epsilon_{2}-\frac{35}{4}\epsilon_{3}$	0.405
110[001]	$1-\frac{21}{10}\epsilon_{1}+\frac{365}{14}\epsilon_{2}+\frac{175}{52}\epsilon_{3}$	0.228
$111[\bar{1}10];111[11\bar{2}]$	$1+\frac{12}{5}\epsilon_{1}-\frac{1280}{63}\epsilon_{2}+\frac{1120}{351}\epsilon_{3}$	0.386

### (c) Application to growth of nickel crystal

#### (i) Free energy for different crystal orientation

The linear system of four equations for interfacial stiffness (the middle column in table 3) can be solved using the computed values of stiffness in MD-simulation due to Rozas & Horbach [57] (right column in table 3). One gets then the following values of averaged interface energy, $\gamma_{0}$ and anisotropic strength coefficients, $\epsilon_{i}(i=1,2,3)$,

5.6 $$\gamma_{0}=0.312\;{\text{J m}}^{-2},\;\epsilon_{1}=9.89\text{\%},\;\epsilon_{2}=-0.13\text{\%}\;\text{and}\;\epsilon_{3}=0.85\text{\%}.$$

These values can be used to compute the interfacial free energies with respect to ⟨100⟩, ⟨110⟩ and ⟨111⟩ crystal orientation for Ni, i.e. $\gamma_{100}$, $\gamma_{110}$ and $\gamma_{111}$, respectively, from the equations shown in table 2. The comparison with the results of works [57,58], and the values of the interfacial free energies obtained by using the average interfacial energy, $\gamma_{0}$ from [59,60] is shown in table 4.

**Table 4.** Comparison between the obtained values of interfacial free energy, $\gamma_{100}$, $\gamma_{110}$ and $\gamma_{111}$, and averaged interfacial free energy, $\gamma_{0}$, for Ni and those found in the literature.

$\gamma_{0}$	$\gamma_{100}$	$\gamma_{110}$	$\gamma_{111}$	source
0.302	0.325	0.310	0.304	[57]
0.2186	0.2322	0.2134	0.2126	[58]
0.3402	0.3846	0.3229	0.3216	[58]
0.25	0.269	0.257	0.252	[59] and present work
0.278	0.299	0.285	0.280	[60] and present work
0.312	0.336	0.321	0.314	present work

#### (ii) Velocity-undercooling relationship

In the case of a pure (chemically one component, elemental) system, the driving force, ΔG, can be given by equation (3.12). Therefore, equation (5.1) yields the following anisotropic interface ‘velocity-undercooling’ relationship

5.7 $$V_{klm}=\mp \frac{\mu_{klm}\Delta T}{\sqrt{1+{\left[\mu_{klm}\Delta T/V_{\phi ,klm}\right]}^{2}}},$$

from which one can identify the expression of coefficients of crystal growth kinetics, $\mu_{klm}$, as

5.8 $$\mu_{klm}=\frac{D_{\phi ,klm}}{\gamma_{klm}}\frac{\Delta H_{f}}{T_{m}},$$

where k, l and m are the Miller indices.

#### (iii) Results of computations

**Figure 2.**
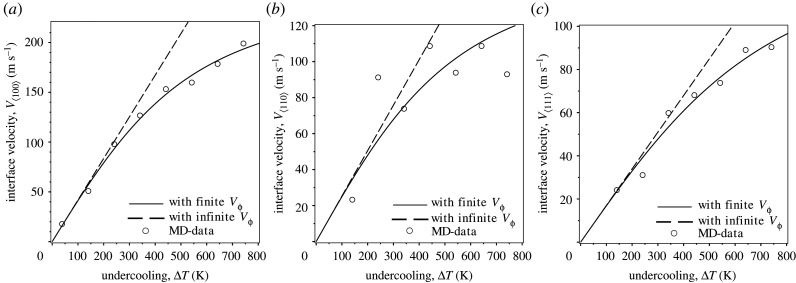
Predictions of nonlinear and linear anisotropic ‘interface velocity-undercooling’-relationship, equation (5.7), i.e. with phase field relaxation time, $\tau_{\phi }\neq 0$, and without phase field relaxation time, $\tau_{\phi }\to 0$, respectively, (continuous line)-and (dashed line) compared with MD-data due to Hoyt *et al.* [47] (**∘**) for Ni. Comparison is made for: (*a*) ⟨100⟩ crystal direction, (*b*) ⟨110⟩ crystal direction and (*c*) ⟨111⟩ crystal direction. The material parameters of Ni used in calculations are shown in table 6.

Using equation (5.7) with equations (5.3)–(5.5) and (5.8), the growth of Ni-crystal in ⟨100⟩, ⟨110⟩ and ⟨111⟩ crystallographic directions can be characterized as follows:

— Knowing the values of interfacial stiffness computed in MD-simulations, the development of the interfacial free energy and its stiffness in cubic harmonics with three strengths of anisotropy, $\epsilon_{i}(i=1,2,3)$, shown in table 2 and table 3, yield a good agreement in comparison with [57,58] and those obtained using the averaged interface energy of [59,60], see table 4.
— Using material parameters of Ni from table 5, figure 2 shows very good predictions of MD-data due to Hoyt *et al.* [47] by the hodograph equation, (3.16), for planar interface, $\kappa_{1}=\kappa_{1}=0$, and constant normal velocity $V_{n}(t)=\text{const}$ given by equation (5.1), where the driving force, ΔG, is given by equation (3.12).
— Table 5 shows the agreement for the obtained values of interface kinetic coefficient, equation (5.8), in comparison with those found in the literature [6,47].
— Using expansion of the interface energy (5.3) and (5.4), we obtained values of kinetic coefficients in three crystallographic directions by the fitting of equations (5.7) and (5.8) to data of MD-simulation. In this respect, one should note that the values of these kinetic coefficients $\mu_{klm}$ correlate well with the values obtained by the authors of the work [47] for the same MD-data, see table 5. This confirms the adequacy of the application of the kinetic equation (5.7) together with (5.8) to the description of anisotropic interfaces in a wide range of undercooling.

**Table 5.** Comparison of the obtained values of interface kinetic coefficient $\mu_{klm}$, equation (5.8), from ‘anisotropic interface velocity-undercooling’ relationship, equation (5.7), for different orientations, i.e. $\mu_{100}$, $\mu_{110}$-and $\mu_{111}$, for Ni and those found in the literature [6,47].

$\mu_{100}$	$\mu_{110}$	$\mu_{111}$	source
0.672	0.586	0.409	[6]
0.719	0.507	0.356	[6]
0.45	0.32	0.18	[47]
0.418	0.253	0.170	present work

**Table 6.** Material parameters of Ni used in calculations.

parameter		value	source
$T_{m}$ (K), melting temperature		1706	[6]
$\Delta S_{f}$ (${\text{J mole}}^{-1}\;{\text{K}}^{-1}$), entropy of fusion		10.73	[60]
$\Delta S_{f}=\frac{\Delta H_{f}}{T_{m}}$ (${\text{J m}}^{-3}\;{\text{K}}^{-1}$), entropy of fusion${\;}^{\text{a}}$		$1.66\times 10^{6}$	present work
$\Delta H_{f}$ (${\text{J m}}^{-3}$), enthalpy of fusion		$2.84\times 10^{9}$	present work
**parameters for (100), (110)-and (111) orientations**			
*γ* (n) (J m^−2^), interfacial free energy^b^			
*γ*100 =0.336	*γ*110 =0.321	*γ*111 =0.314	present work
$D_{\phi }(\text{n})$ (${\text{m}}^{2}\;{\text{s}}^{-1}$), phase field diffusion coefficient${\;}^{\text{c}}$			
$D_{\phi ,100}=8.44\times 10^{-8}$	$D_{\phi ,110}=4.87\times 10^{-8}$	$D_{\phi ,111}=3.2\times 10^{-8}$	present work
$V_{\phi }(\text{n})$ (${\text{m s}}^{-1}$), maximum phase field speed${\;}^{\text{d}}$			
$V_{\phi ,100}=247.3$	$V_{\phi ,110}=152.1$	$V_{\phi ,111}=136.8$	present work

${\;}^{\text{b}}$the values of interfacial free energy used in fit for different orientations fromtable 4.

${\;}^{\text{c}}$the values of phase field diffusion coefficient, $D_{\phi }(n)$, obtained in this work from fit for different orientations.

${\;}^{\text{a}}$the enthalpy of fusion, $\Delta H_{f}$, is calculated through the entropy of fusion, $\Delta S_{f}$, due to Jian *et al.* [60] taking into account the melting temperature, $T_{m}$, due to Mendelev *et al.* [6].

Note that for each ⟨100⟩, ⟨110⟩ and ⟨111⟩ orientations with the values of interfacial free energies $\gamma_{100}$, $\gamma_{110}$ and $\gamma_{111}$, the melting temperature, $T_{m}$ and the enthalpy of fusion, $\Delta H_{f}$, respectively (table 6), the phase field diffusion $D_{\phi ,100}$, $D_{\phi ,110}$ and $D_{\phi ,111}$, and the maximum speeds of phase field propagation, $V_{\phi ,100}$, $V_{\phi ,110}$ and $V_{\phi ,111}$, which are considered as parameters, are obtained by fitting MD-data due to Hoyt *et al.* [47]. These latter are shown in the 10th and 12th rows of table 6, respectively.

## 6. Conclusion

The present work has been devoted to the derivation and analysis of the slow and fast interface propagation described by the hodograph equation following from the phase field model. The obtained solution of the hodograph equation describes the amplitude, width and velocity of the anisotropic interface in the slow and fast regimes of dynamics. We show that the obtained hodograph equation is consistent with classical equations of the Herring–Gibbs–Thomson-type as well as the velocity-dependent Herring equation for crystal growth, Klein–Gordon equation for the front’s propagation of the inflation stage of matter, and Born–Infeld equation of the nonlinear electrodynamics. The first benchmarks of the derived hodograph equation of anisotropic interface motion show the consistency of its solution with the steady-state growth of nickel crystals obtained for different crystallographic directions using molecular dynamics simulation of Hoyt *et al.* [47].

## Data accessibility

Electronic supplementary material on asymptotic analysis of the hyperbolic phase field model are attached to the main text of the manuscript.

## Appendix A. Derivation of the phase field equation from Lyapunov’s stability condition

The temporal derivative of the free energy functional equation (3.4) is described by

A 1 $$\begin{array}{rl} \frac{\text{d}G}{\text{d}t} & =\int_{v_{0}}\frac{\partial }{\partial t}\left\{\frac{1}{2}{\left[\gamma (-\nabla \phi )\right]}^{2}+G(T,C,\phi ,\partial \phi /\partial t)\right\}\text{d}v_{0}\\ & =\int_{v_{0}}\gamma (-\nabla \phi )\frac{\partial (\gamma (-\nabla \phi ))}{\partial t}\;\text{d}v_{0}+\int_{v_{0}}\frac{\partial G(T,C,\phi ,\partial \phi /\partial t)}{\partial t}\;\text{d}v_{0}, \end{array}$$

where ϕ is the phase field (non-conserved order parameter) and other notations are given in table 1. Then, the first integral in equation (A 1) gives

A 2 $$\begin{array}{rl} & \int_{v_{0}}\gamma (-\nabla \phi )\frac{\partial (\gamma (-\nabla \phi ))}{\partial t}\;\text{d}v_{0}\\ & \;=\int_{v_{0}}\gamma (-\nabla \phi )\frac{\partial (\gamma (-\nabla \phi ))}{\partial (\nabla \phi )}\cdot \frac{\partial (\nabla \phi )}{\partial t}\;\text{d}v_{0}\\ & \;=\int_{v_{0}}\nabla \cdot \left(\frac{\partial \phi }{\partial t}\gamma (-\nabla \phi )\frac{\partial (\gamma (-\nabla \phi ))}{\partial (\nabla \phi )}\right)\;\text{d}v_{0}-\int_{v_{0}}\frac{\partial \phi }{\partial t}\nabla \cdot \left(\gamma (-\nabla \phi )\frac{\partial (\gamma (-\nabla \phi ))}{\partial (\nabla \phi )}\right)\;\text{d}v_{0}\\ & \;=-\int_{v_{0}}\frac{\partial \phi }{\partial t}\nabla \cdot \left(\gamma (-\nabla \phi )\frac{\partial (\gamma (-\nabla \phi ))}{\partial (\nabla \phi )}\right)\;\text{d}v_{0}, \end{array}$$

where the following integration is taken into account

A 3 $$\int_{v_{0}}\nabla \cdot \left[\frac{\partial \phi }{\partial t}\left(\gamma (-\nabla \phi )\frac{\partial (\gamma (-\nabla \phi ))}{\partial (\nabla \phi )}\right)\right]\text{d}v_{0}=\int_{\Gamma }\frac{\partial \phi }{\partial t}\left(\gamma (-\nabla \phi )\frac{\partial (\gamma (-\nabla \phi ))}{\partial (\nabla \phi )}\right)\cdot n_{0}\;\text{d}\Gamma =0,$$

with $n_{0}$ the normal vector to the outer surface Γ of the volume $v_{0}$.

Using the definition of the free energy [38]

A 4 $$G_{\text{eq}}(T,C,\phi )=[1-p(\phi )]G_{l}(T,C)+p(\phi )G_{s}(T,C)+W_{\phi }(T,C)g(\phi )+\frac{\alpha_{\phi }(T,C)}{2}{\left(\frac{\partial \phi }{\partial t}\right)}^{2},$$

with g(ϕ) the double-well function and p(ϕ) the interpolation function given by equation (2.5), the time derivative of G(T,C,ϕ,∂ϕ/∂t) can be written as

A 5 $$\begin{array}{rl} \frac{\partial G(T,C,\phi ,\partial \phi /\partial t)}{\partial t} & =\left[[1-p(\phi )]\frac{\partial G_{l}(T,C)}{\partial T}+p(\phi )\frac{\partial G_{s}(T,C)}{\partial T}+g(\phi )\frac{\partial W_{\phi }(T,C)}{\partial T}\right]\frac{\partial T}{\partial t}\\ & \;+\left[[1-p(\phi )]\frac{\partial G_{l}(T,C)}{\partial C}+p(\phi )\frac{\partial G_{s}(T,C)}{\partial C}+g(\phi )\frac{\partial W_{\phi }(T,C)}{\partial C}\right]\frac{\partial C}{\partial t}\\ & \;+\left[\Delta G\frac{\partial p(\phi )}{\partial \phi }+W_{\phi }(T,C)\frac{\partial g(\phi )}{\partial \phi }+\alpha_{\phi }\frac{\partial^{2}\phi }{\partial t^{2}}\right]\frac{\partial \phi }{\partial t}, \end{array}$$

where $\Delta G=G_{s}-G_{l}$ is the driving force which has the form of Gibbs free energy change on solidification (ΔG<0) or melting (ΔG>0). Together with equation (A 2), this derivative leads to

A 6 $$\begin{array}{rl} \frac{dG}{dt} & =\int_{V}\{\left[[1-p(\phi )]\frac{\partial G_{l}(T,C)}{\partial T}+p(\phi )\frac{\partial G_{s}(T,C)}{\partial T}+g(\phi )\frac{\partial W_{\phi }(T,C)}{\partial T}\right]\frac{\partial T}{\partial t}\\ & \;+\left[[1-p(\phi )]\frac{\partial G_{l}(T,C)}{\partial C}+p(\phi )\frac{\partial G_{s}(T,C)}{\partial C}+g(\phi )\frac{\partial W_{\phi }(T,C)}{\partial C}\right]\frac{\partial C}{\partial t}\\ & \;+\left[\Delta G\frac{\partial p(\phi )}{\partial \phi }+W_{\phi }(T,C)\frac{\partial g(\phi )}{\partial \phi }+\alpha_{\phi }\frac{\partial^{2}\phi }{\partial t^{2}}-\nabla \cdot \left(\gamma (-\nabla \phi )\frac{\partial (\gamma (-\nabla \phi ))}{\partial (\nabla \phi )}\right)\right]\frac{\partial \phi }{\partial t}\}\text{d}V. \end{array}$$

Now we apply the Lyapunov stability condition according to which the evolving system must have non-increasing free energy in time. For the phase field model, the condition should be taken as [38]: dG/dt≤0, that gives for equation (A 6) the following system of equations:

A 7 $$\begin{array}{rl} & \frac{\partial T}{\partial t}=-M_{T}[[1-p(\phi )]\frac{\partial G_{l}(T,C)}{\partial T}+p(\phi )\frac{\partial G_{s}(T,C)}{\partial T}+g(\phi )\frac{\partial W_{\phi }(T,C)}{\partial T}], \end{array}$$

A 8 $$\begin{array}{rl} & \frac{\partial C}{\partial t}=-M_{C}[[1-p(\phi )]\frac{\partial G_{l}(T,C)}{\partial C}+p(\phi )\frac{\partial G_{s}(T,C)}{\partial C}+g(\phi )\frac{\partial W_{\phi }(T,C)}{\partial C}] \end{array}$$

A 9 $$\begin{array}{rl} \;\text{and}\; & \frac{\partial \phi }{\partial t}=-M_{\phi }[\Delta G\frac{\partial p(\phi )}{\partial \phi }+W_{\phi }(T,C)\frac{\partial g(\phi )}{\partial \phi }+\alpha_{\phi }\frac{\partial^{2}\phi }{\partial t^{2}}-\nabla \cdot (\gamma (-\nabla \phi )\xi )], \end{array}$$

with ξ-vector is defined by equation (3.10), and $M_{T}>0,$ $M_{C}>0$ and $M_{\phi }>0$ are positive coefficients to ensure the condition dG/dt≤0. These coefficients define the intensity of heat transfer, mass transport and dynamics of the interface motion, respectively.

Using the definition of relaxation time $\tau_{\phi }=M_{\phi }\alpha_{\phi }$, equation (A 9) leads to

A 10 $$\tau_{\phi }\frac{\partial^{2}\phi }{\partial t^{2}}+\frac{\partial \phi }{\partial t}=M_{\phi }\nabla \cdot (\gamma (-\nabla \phi )\xi )-M_{\phi }\left[\Delta G\frac{\partial p(\phi )}{\partial \phi }+W_{\phi }(T,C)\frac{\partial g(\phi )}{\partial \phi }\right].$$

where the additional constraint, which follows from equations (A 7) and (A 8) for the isothermal conditions, T≡const, and stationary concentration, ∂C/∂t=0, are given by

A 11 $$[1-p(\phi )]\frac{\partial G_{l}(T,C)}{\partial T}+p(\phi )\frac{\partial G_{s}(T,C)}{\partial T}+g(\phi )\frac{\partial W_{\phi }(T,C)}{\partial T}=0$$

and

A 12 $$[1-p(\phi )]\frac{\partial G_{l}(T,C)}{\partial C}+p(\phi )\frac{\partial G_{s}(T,C)}{\partial C}+g(\phi )\frac{\partial W_{\phi }(T,C)}{\partial C}=0.$$

Note that the above additional constraint, equations (A 11)–(A 12), reduces to equation (A 11) for the one-component system, C=0, under isothermal conditions, T=const.
